# Evaluating Software Tools for Lipid Identification from Ion Mobility Spectrometry–Mass Spectrometry Lipidomics Data

**DOI:** 10.3390/molecules28083483

**Published:** 2023-04-14

**Authors:** Dylan H. Ross, Jian Guo, Aivett Bilbao, Tao Huan, Richard D. Smith, Xueyun Zheng

**Affiliations:** 1 Earth and Biological Sciences Directorate, Pacific Northwest National Laboratory, Richland, WA 99354, USA; 2 Department of Chemistry, Faculty of Science, University of British Columbia, Vancouver Campus, 2036 Main Mall, Vancouver, BC V6T 1Z1, Canada

**Keywords:** software, ion mobility spectrometry, mass spectrometry, lipidomics, lipid identification

## Abstract

The unambiguous identification of lipids is a critical component of lipidomics studies and greatly impacts the interpretation and significance of analyses as well as the ultimate biological understandings derived from measurements. The level of structural detail that is available for lipid identifications is largely determined by the analytical platform being used. Mass spectrometry (MS) coupled with liquid chromatography (LC) is the predominant combination of analytical techniques used for lipidomics studies, and these methods can provide fairly detailed lipid identification. More recently, ion mobility spectrometry (IMS) has begun to see greater adoption in lipidomics studies thanks to the additional dimension of separation that it provides and the added structural information that can support lipid identification. At present, relatively few software tools are available for IMS-MS lipidomics data analysis, which reflects the still limited adoption of IMS as well as the limited software support. This fact is even more pronounced for isomer identifications, such as the determination of double bond positions or integration with MS-based imaging. In this review, we survey the landscape of software tools that are available for the analysis of IMS-MS-based lipidomics data and we evaluate lipid identifications produced by these tools using open-access data sourced from the peer-reviewed lipidomics literature.

## 1. Introduction

Lipids are essential biomolecules in numerous cellular processes, and their unambiguous identification and comprehensive structure elucidation can increase our understanding of their functions and their use as biosignatures [1,2,3]. Advances in the field of lipidomics can be largely attributed to advances in mass spectrometry (MS) and analytical separations [4]. Currently, most MS-based lipidomics characterization is performed via direct sample infusion or in conjunction with on-line liquid chromatographic separation (LC-MS). Both approaches are easily combined with tandem MS/MS, which can identify the fatty acyl chains and the head groups present for each detected lipid. However, the analysis of lipids is still challenging due to their complex and diverse chemical structures, which often comprise numerous isomeric structures originating from different fatty acyl chain lengths, fatty acyl positions, and C=C double bond orientations (cis vs. trans) and locations.

Ion mobility spectrometry (IMS) provides an additional dimension of separation based on the shape and size of the analyte ions, where their measured mobility can be converted to collision cross section (CCS) [5,6,7]. IMS is particularly attractive for lipidomics as it allows for the distinguishing of lipid classes in many cases, as well as the separation of isomers [8,9,10,11,12,13,14,15]. Furthermore, when combined as an integrated LC-IMS-MS workflow, the three-dimensional separations with LC elution times, IMS drift times (or CCS values), and *m*/*z* ratios provide a basis for the exhaustive characterization of lipids in complex samples (e.g., tissue) [16,17,18]. MS/MS information is usually necessary for determining the composition of individual fatty acids for lipids containing multiple acyl chains. MS/MS data were acquired using one of two techniques: data-dependent acquisition (DDA), in which only selected ions are fragmented, or data-independent acquisition (DIA), in which all ions are fragmented without prior selection. MS/MS with many widely used IMS instruments (i.e., classic drift tube IMS (DTIMS) [6,19] and traveling wave IMS (TWIMS) [20] platforms) is generally performed in a CID cell where all the ions are fragmented after the IMS separation (i.e., operated in a DIA mode). Recently, the trapped IMS (TIMS) platform was reported to enable effective DDA MS/MS using a parallel accumulation–serial fragmentation (PASEF) technique that synchronizes the TIMS separation with MS/MS precursor selection [15].

While the advancement of IMS technology and experimental strategies with MS have greatly improved the in-depth characterization and identification of lipids, analysis of IMS-MS data which includes multidimensional separations has been challenging and has limited the full utility of these measurements, especially for complex samples.

In this minireview, we survey the software landscape and the capabilities for lipid identification using existing tools for IMS-MS lipidomics using published datasets based upon the use of well-characterized lipid extracts. In particular, we consider different analysis workflows for LC-IMS-MS/MS using two MS/MS approaches, DIA vs. DDA, for lipid identifications of the total lipid extracts from the NIST 1950 SRM plasma.

## 2. Lipidomics Data Analysis

**Figure 1:**
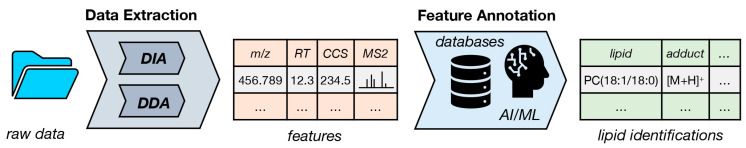
Generalized workflow for analysis of IMS-MS-based lipidomics data. Starting from raw data, either in original vendor format or converted to another format, the first step involves the extraction and processing of data to produce features. These features are defined by the measurement dimensions in the experiment, most commonly *m*/*z*, retention time (RT), IMS collision cross section (CCS), and MS/MS spectra (MS2) for LC-IMS-MS/MS experiments. The next step in the process is to assign lipid annotations to the extracted features using one or more of the measured properties of the features. Reference values for these properties may come from databases of previously measured values or predictions based on theoretical principles or empirically derived trends. The final result is a list of extracted features with associated lipid annotations.

The analysis of lipidomics data generally involves two major processes: data extraction and feature annotation (Figure 1). Data extraction consists of extracting and processing signals from raw data and constructing features from measurement values. In this context, a feature corresponds to a collection of measured values from all separation dimensions in the analytical approach (for instance, an LC-MS feature is defined by retention time from the LC dimension and *m*/*z* and intensity from the MS dimension). The specific steps for data extraction are dependent upon the details of the analytical approach, and this is particularly the case for experiments including MS/MS data acquired using DIA vs. DDA. Feature annotation describes the process of comparing a feature’s measured values to reference values for lipid annotations. These reference values may be sourced from previously made measurements and/or values predicted on the basis of theory or empirical trends. The confidence of lipid annotations, often reflected by a scoring metric, is derived from the degree of similarity between measurements and reference values. Different software tools perform part or all of these steps, and the precise order of operations can vary according to the requirements or constraints of a particular experimental design. Moran-Garrido et al. have recently published a review that delves more deeply into the details of lipidomics data analysis as it relates to specific instrumentation and acquisition methods [21].

## 3. Survey of Existing Lipidomics Data Analysis Software

We surveyed the bioinformatic programs which facilitate part or all of the workflow for processing IMS-MS-based lipidomics data. The characteristics of all the free-to-use programs are summarized in Table 1. The tools in the table are organized according to function, specifically reflecting which tools perform feature extraction and/or annotation. Skyline [22] and MS-DIAL [23] cover the whole data processing workflow including the feature extraction and lipid identification. LiPydomics [24], LipidIMMS (Lipid4DAnalyzer) [25], and AllCCS [26] only perform lipid identification, whereas MZmine3 [27] and DEIMoS [28] only perform feature extraction. MZmine3 can perform annotation (local compound database search) by RT and CCS given a user-provided database in csv format. PNNL PreProcessor [29] can be used for preprocessing the data before feature extraction, making it distinct from the others. Regarding the supported data formats, most programs can process multiple vendors’ data formats either directly or after conversion to an intermediate format. Though not included in Table 1 due to being proprietary software, Lipid Annotator [30], Lipostar [31], and MetaboScape (Bruker Daltonics, Billerica, MA, USA) are specialized tools that can process IMS-MS lipidomics data from Agilent, Waters, and Bruker platforms, respectively. MS-DIAL requires the files to be converted to an IBF format, whereas Skyline can directly process files in raw vendor formats. Skyline is distinctive as it is the only program in this list that performs targeted feature extraction and lipid identification. All programs but MZmine3 facilitate MS2 deconvolution and can process DIA data. With regard to calibration, Skyline and LipidIMMS perform RT calibration; LiPydomics performs RT calibration for HILIC separations; AllCCS and DEIMoS can perform CCS calibration; and Skyline, MS-DIAL, and MZmine3 can read the CCS calibration performed by the instrument vendor software. AllCCS is the only tool that does not generate any quantitative results. As for the lipid identification, all the programs capable of performing lipid identification have their own library containing MS2 spectra, RT, and CCS information. The information in the library is experimental, in silico, or hybrid. The lipid identifications produced by all of the discussed tools are at the molecular species level, consisting of the lipid class and the number of carbons and double bonds in each fatty acid chain.

## 4. Evaluation of Lipidomics Data Analysis Software

### 4.1. Selection of Evaluation Data

In order to evaluate the software tools that are currently available for processing IMS-MS lipidomics data, we sought out existing publicly available datasets with published (and peer-reviewed) analysis results. We constrained our search of the literature to published datasets that included NIST SRM-1950 plasma because considerable efforts toward standardization of lipidomics analyses using this standard reference material have been published previously [32,33]. As a result, the lipid composition of this material has already been well characterized, providing a basis for the comparison of lipid identifications from evaluations using different software tools. An additional consideration in our dataset search was the analytical platform used for data acquisition. Specifically, we sought IMS-MS lipidomics datasets acquired using each of the major IMS-MS platforms in the field (i.e., DTIMS, TWIMS, TIMS), ideally with coverage of MS/MS acquisition (DIA and DDA) and ionization (positive and negative) modes. Ultimately, accounting for all of the above considerations, we were only able to find two suitable published datasets for our evaluation of software tools: an LC-DTIMS-MS/MS dataset from Kirkwood et al. (2022) [34] and an LC-TIMS-MS/MS dataset from Vasilopoulou et al. (2020) [15]. The LC-DTIMS-MS/MS dataset was acquired using RPLC (using an approximately 30 min solvent gradient) coupled to an Agilent 6560 DTIMS-qTOF mass spectrometer operated with post-mobility All Ion MS/MS (DIA) for three replicates of SRM plasma in both positive and negative ionization modes. The LC-TIMS-MS/MS dataset was acquired using RPLC coupled to a Bruker timsTOF Pro mass spectrometer operated with parallel accumulation–serial elution and fragmentation MS/MS (PASEF, DDA) for five replicates of SRM plasma in both positive and negative ionization modes. These datasets representing two different data acquisition strategies (DDA vs. DIA) come from two of the three major analytical platforms widely used in the field and include multiple technical replicates acquired in both positive and negative ionization modes, making them the best suited among the available data for evaluating the current software landscape for the analysis of IMS-MS lipidomics data.

### 4.2. Selection of Software Tools for Evaluation

In the previous section, we comprehensively surveyed the software landscape for the analysis of IMS-MS lipidomics data. Although this landscape is significantly less broad than that for general lipidomics data analysis [35], there are still too many tools for systematic evaluation to be practical. We therefore settled on two software tools to evaluate using the selected SRM plasma data: Skyline [22] and MS-DIAL [23]. We chose these tools because (1) they are free and open-source, (2) they perform the complete lipidomics data analysis process from data extraction to feature annotation, and (3) they both support data from the two platforms used to acquire the evaluation data we selected.

### 4.3. Analysis of LC-DTIMS-MS/MS Data Using Skyline and MS-DIAL

To evaluate Skyline for the analysis of LC-DTIMS-MS/MS lipidomics data, we followed the protocol provided in Kirkwood et al. (2022) [22]. Briefly, we downloaded and installed the latest version of Skyline (22.2), and the small molecule interface was selected. Then, the library files *2_Plasma_Lipid_Library_Positive.sky.zip* and *2_Plasma_Lipid_Library_Negative.sky.zip* were downloaded from the website specified in the protocol (https://panoramaweb.org/baker-lipid-ims.url, accessed on 1 February 2022). All parameters for data processing were set according to specifications in the protocol, and then the raw data were imported into Skyline for automated data processing. Because Skyline performs targeted feature extraction, data processing proceeds quickly with each file taking only 1–2 min to process. Lipids are identified based on the precursor mass, retention time, and CCS matching from the library. The MS2 spectra are necessary to confirm the number of carbons and double bonds of individual fatty acid chains; therefore, manual validation of the identified lipids by checking the chromatographic peak shape, the isotopic pattern, and the quality of the MS2 spectra is required to avoid false positive identifications. If the chromatographic peak shape was noisy, did not appear in all three replicates, or the isotopic pattern differed significantly from the expected pattern for the lipid annotation, the lipid target was removed. After manual verification, the final list of identified lipids was exported as a *.csv* file. In total, 217 and 223 lipids were identified from the positive and negative mode data, respectively, for a total of 440 identifications, which is similar to but slightly less than the total of 483 reported in the source publication [34]. We believe the primary source of this discrepancy between the total number of identifications comes from the process of manually verifying peak shapes, isotopic patterns, and quality of MS2 spectra, which inherently introduces some level of bias in the results. However, despite this slight discrepancy between the number of lipids identified, the categories of lipids identified in the present work are qualitatively similar to those in the source publication and they generally comport with previous in-depth studies on the composition of SRM plasma [32,33].

We also evaluated MS-DIAL for the analysis of LC-DTIMS-MS/MS lipidomics data. Briefly, we downloaded and installed a recent version of MS-DIAL (4.92). Then, the raw data were converted to IBF format using *ibfConverter* provided with the program. MS-DIAL was opened, a new project was created under the folder containing the IBF files, and ion mobility was selected in the separation type section. Data-independent MS/MS was selected in the MS method type section. Centroid data were selected for both MS1 and MS/MS. Positive or negative was selected in the ion mode depending on whether the positive or negative data set was being processed. Lipidomics was selected in the target omics section. The default analysis parameters were retained. In the identification tab, MSP file was selected, and all lipids were checked. In the alignment tab, the “100% should be detected in all replicated samples” option was specified. Because MS-DIAL performs untargeted feature extraction which includes MS2 deconvolution, data processing takes much longer than with Skyline (over two days to process the three replicates in positive and negative ionization modes). As with Skyline, MS-DIAL performs lipid identification based on similarity between precursor mass, CCS, and MS2 spectra for detected features and its lipid database. To avoid false positive identifications, we manually verified the results using the same criteria as described above for Skyline. The final results were exported as a *.csv* file. In total, 223 and 49 lipids were identified from the positive and negative mode data, respectively. The total number of lipids identified (272) is lower than the total from Skyline, and this difference is primarily attributable to there being significantly less identifications from the negative mode data. Specifically, MS-DIAL identifies much fewer FA and PC than Skyline from the negative mode data. This discrepancy could be attributable to a lack of coverage for these classes in the negative mode in the internal database that MS-DIAL uses to identify lipids. It is also possible that the weighting of the contributing factors for making a lipid identification (i.e., retention time, isotope pattern, MS/MS spectral matching) differs between MS-DIAL and Skyline, and those differences manifest as systematic differences in the lipid annotations they produce.

**Figure 2:**
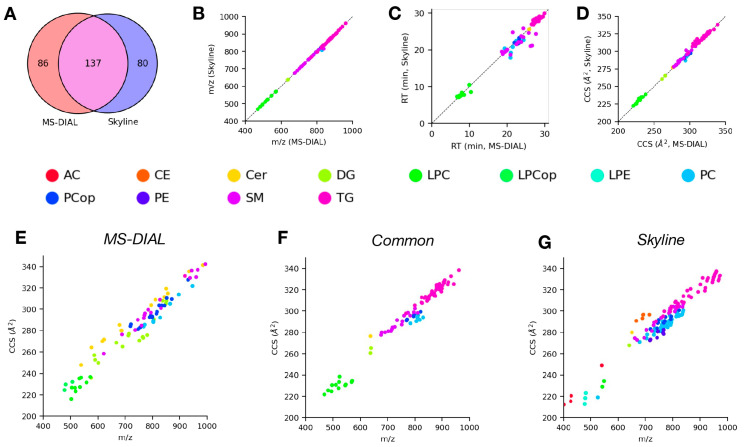
Comparison of lipid identifications from analysis of positive mode LC-DTIMS-MS/MS lipidomics data using Skyline and MS-DIAL. (**A**) Venn diagram of lipids identified using either software. (**B**–**D**) Comparisons of *m*/*z* (**B**), retention time (**C**), and CCS (**D**) values from MS-DIAL vs. Skyline for common lipid identifications from both tools, points colored according to lipid class. (**E**,**F**) CCS trend lines or IMS-MS conformational space of lipids identified by MS-DIAL (**E**), Skyline (**G**), or both tools (**F**), points colored according to lipid class.

**Figure 3:**
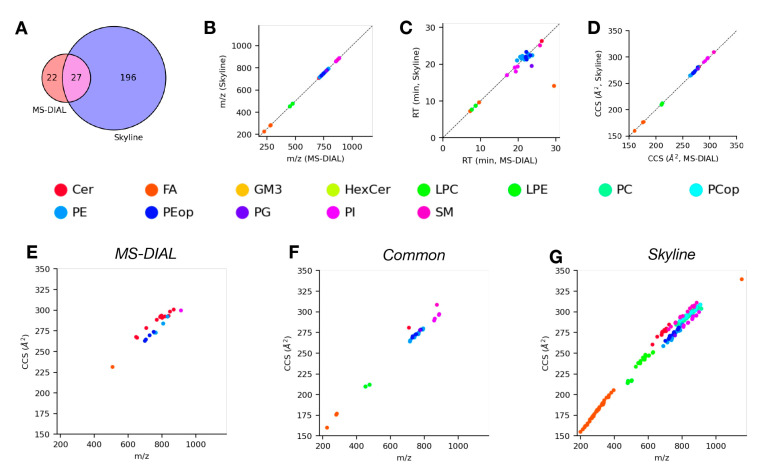
Comparison of lipid identifications from analysis of negative mode LC-DTIMS-MS/MS lipidomics data using Skyline and MS-DIAL. (**A**) Venn diagram of lipids identified using either software. (**B**–**D**) Comparisons of *m*/*z* (**B**), retention time (**C**), and CCS (**D**) values from MS-DIAL vs. Skyline for common lipid identifications from both tools, points colored according to lipid class. (**E**,**F**) CCS trend lines or IMS-MS conformational space of lipids identified by MS-DIAL (**E**), Skyline (**G**), or both tools (**F**), points colored according to lipid class.

We next compared the lipid identifications from Skyline and MS-DIAL for the positive (Figure 2) and negative (Figure 3) mode data discussed above to gain insight on how similar the results are when analyzing the same data using different software. Figure 2A shows the high degree of overlap between lipid identifications from Skyline and MS-DIAL for the positive mode data. Specifically, there were 137 common lipids identified between the two tools, with 86 and 80 lipids only identified in MS-DIAL or Skyline, respectively. Among the common lipid identifications, there was generally a high degree of agreement between the corresponding measurement values (*m*/*z*, Figure 2B; RT, Figure 2C; CCS, Figure 2D) from both tools across all of the observed lipid classes. The largest amount of variability was observed for RT, which makes sense given the often noisy nature of chromatographic profiles and differences in signal processing and fitting methods between the tools. We also examined plots of CCS vs. *m*/*z*, commonly referred to as the CCS trend line or “IMS-MS conformational space”, for lipids identified only by either tool individually (Figure 2E,G) or identified by both tools (Figure 2F) to assess lipid class coverage in addition to the overall reasonability of lipid identifications based on their trends in this space [36]. The common lipid identifications consist primarily of the TG, SM, and PC/LPC lipid classes, all of which generally follow the expected characteristic trends in the IMS-MS conformational space. The lipids only identified by MS-DIAL mostly consist of the PE, Cer, and DG lipid classes, whereas those only identified by Skyline are predominantly TG, PC, and PE lipid classes. Figure 3A shows the degree of overlap between lipids identified by MS-DIAL and Skyline for the negative mode data. Although the very small number of identifications from MS-DIAL limit what can be taken away from this comparison, we observed similar trends among the commonly identified lipids with respect to measured properties (Figure 3B–D) as we did for the positive mode data. Most lipids identified from the negative mode data came from Skyline, and therefore the distribution of these lipid identifications in IMS-MS conformational space (Figure 3G) is most interesting for this set of results. We observed a diverse range of lipid classes, each following distinct trends in this conformational space owing to the unique structural properties of each lipid class. Taken together, these observations from the comparison between lipids identified from DTIMS lipidomics data analysis using Skyline and MS-DIAL in positive mode demonstrate that both tools produce similar results at a high level. A comparison of the negative mode results was not possible due to an unidentified seemingly systemic error with the ability of MS-DIAL to process the negative mode DTIMS data. More specifically, the primary source of this discrepancy seemed to be a lack of identifications for the FA and PC classes, but we were unable to determine the cause. For the positive mode data, at a more granular level, there are specific and systematic differences between the lipids that are identified using these tools, and these differences are likely attributable to (1) the specific details of how each tool extracts and processes data, (2) the data sources and methods of constructing the internal databases that the tools use for making lipid identifications, and (3) biases introduced by the user through the manual verification of the initial results produced by the tools.

### 4.4. Analysis of LC-TIMS-MS/MS Data Using Skyline and MS-DIAL

We performed a similar evaluation as described above for Skyline and MS-DIAL but using LC-TIMS-MS/MS lipidomics data [15]. The process for data analysis using Skyline was the same as outlined above, except for the added step of adjusting the transition settings so that the acquisition mode was set to DDA prior to data processing. Likewise with the MS-DIAL data analysis, the procedure was the same as described above except that the MS/MS method parameter was set to DDA prior to data processing. Data processing with Skyline proceeded quickly (1–2 min per replicate), as was the case for the DTIMS data. The data processing with MS-DIAL was much faster (<1 h per replicate) for this data than it was for the DTIMS data, likely due to this data being acquired in a DDA acquisition mode and therefore not requiring computationally expensive deconvolution. In total, 202 and 238 lipids were identified using MS-DIAL from the positive and negative mode data, respectively (440 total). A total of 101 and 154 lipids were identified using Skyline from the positive and negative mode data, respectively (256 total). The total identifications from Skyline were considerably lower than the total from MS-DIAL, which we primarily attribute to an apparent inability of Skyline to properly extract MS2 spectra from this TIMS-PASEF DDA data, which we were unable to fix despite trying many combinations of parameter settings. It is not clear at this time whether this issue is related to Skyline itself or the library/transition settings being used. We could not directly compare the total numbers of lipid identifications from this evaluation to the totals in the source publication because a different (and proprietary) software tool was used to analyze the data and the original identifications in the source publication have been subject to some discussion in the literature [15,37,38]. However, the 440 total lipid identifications from MS-DIAL is in line with the amount of identifications that are made from SRM plasma on many platforms, and the qualitative profile of lipid categories is similar to previous in-depth studies on the composition of SRM plasma [32,33].

**Figure 4:**
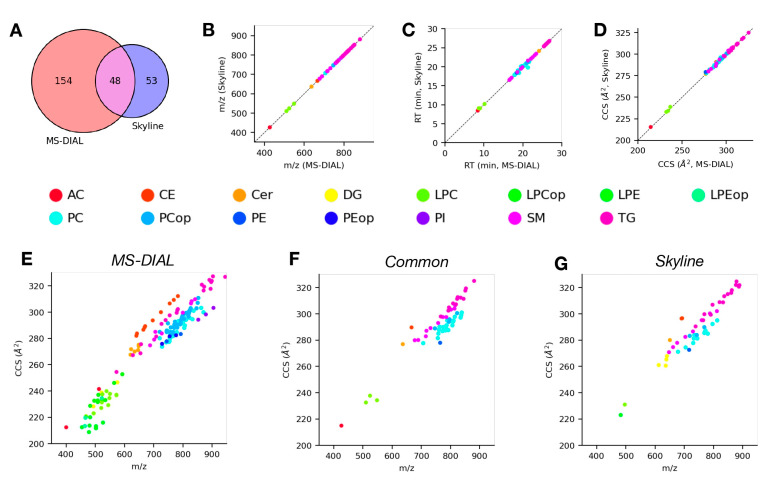
Comparison of lipid identifications from analysis of positive mode LC-TIMS-MS/MS lipidomics data using Skyline and MS-DIAL. (**A**) Venn diagram of lipids identified using either software. (**B**–**D**) Comparisons of *m*/*z* (**B**), retention time (**C**), and CCS (**D**) values from MS-DIAL vs. Skyline for common lipid identifications from both tools, points colored according to lipid class. (**E**,**F**) CCS trend lines or IMS-MS conformational space of lipids identified by MS-DIAL (**E**), Skyline (**G**), or both tools (**F**), points colored according to lipid class.

**Figure 5:**
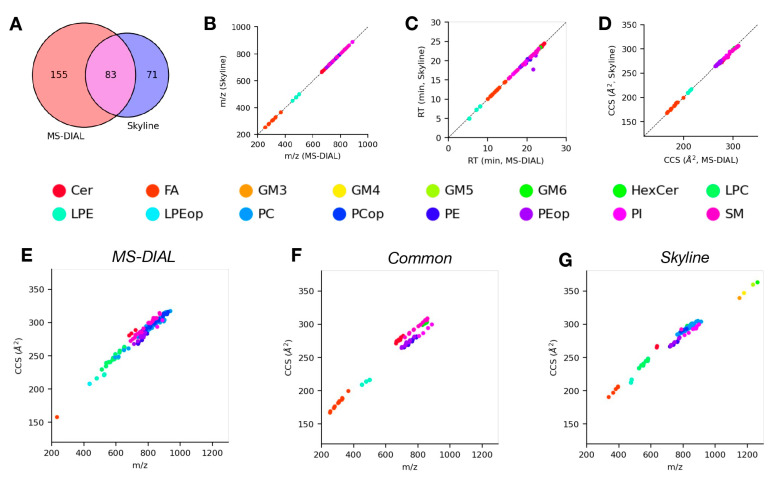
Comparison of lipid identifications from analysis of negative mode LC-TIMS-MS/MS lipidomics data using Skyline and MS-DIAL. (**A**) Venn diagram of lipids identified using either software. (**B**–**D**) Comparisons of *m*/*z* (**B**), retention time (**C**), and CCS (**D**) values from MS-DIAL vs. Skyline for common lipid identifications from both tools, points colored according to lipid class. (**E**,**F**) CCS trend lines or IMS-MS conformational space of lipids identified by MS-DIAL (**E**), Skyline (**G**), or both tools (**F**), points colored according to lipid class.

Just as with the LC-DTIMS-MS/MS dataset, we compared lipid identifications made using Skyline and MS-DIAL for this evaluation dataset in both positive (Figure 4) and negative (Figure 5) ionization modes. Figure 4A shows a modest degree of overlap between lipid identifications from Skyline and MS-DIAL for the positive mode data. Specifically, there were 48 common lipids identified between the two tools, with 154 and 53 lipids only identified in MS-DIAL or Skyline, respectively. Among the common lipid identifications, there was a high degree of agreement between the corresponding measurement values (*m*/*z*, Figure 4B; RT, Figure 4C; CCS, Figure 4D) from both tools across all of the observed lipid classes, with none of the measurement dimensions displaying significant differences. The IMS-MS conformational space for lipid identifications from either tool individually (Figure 4E,G) and common identifications (Figure 4F) were again used to assess lipid class coverage in addition to their structural trends. Similar to the results for positive mode DTIMS data, the common lipid identifications consist primarily of the TG, SM, and PC lipid classes, all of which follow expected trends in this space. The lipids only identified by MS-DIAL span a wide variety of lipid classes including SM, PC/LPC, PE, and CE. The lipids only identified by Skyline are predominantly TG and PC lipid classes. As was the case for positive mode identifications, Figure 5A shows moderate overlap between lipid identifications from Skyline and MS-DIAL for the negative mode data. Specifically, there were 83 common lipids identified between the two tools, with 155 and 71 lipids only identified in MS-DIAL or Skyline, respectively. We observed similar trends among the commonly identified lipids with respect to measured properties (Figure 5B–D) as we did from the positive mode data. Looking at the distribution of common lipid identifications in the IMS-MS conformational space (Figure 5F), we can see that a large variety of lipid classes, including Cer, FA, PE, and SM, are identified using both tools and their structural trends are consistent with expectations. The lipids only identified using MS-DIAL (Figure 5E) or Skyline (Figure 5G) also cover a wide variety of lipid classes, with PI, SM, PE, and LPC being dominant among the identifications from both tools. As we observed with the DTIMS results, at a high level, the lipid identifications from Skyline and MS-DIAL in these data do not differ very greatly. In this evaluation, the results are similar even at a more granular level; however, systematic differences still arise, and these are attributable to the same factors as discussed for the DTIMS data above.

## 5. Discussion and Future Outlook

We have reviewed the current software landscape for the analysis of IMS-MS lipidomics data and performed in-depth evaluations of two important tools using lipidomics data from two well-established experimental platforms. Despite the current and ever-increasing interest in IMS-MS lipidomics, the software landscape for data analysis (especially free and open-source software) is surprisingly narrow. Indeed, only two free and open-source tools, Skyline and MS-DIAL, are capable of performing end-to-end analysis (data extraction and lipid identification) of IMS-MS lipidomics data. Using published data acquired using LC-DTIMS-MS/MS and LC-TIMS-MS/MS platforms for SRM-1950 plasma, we evaluated the lipid identifications from MS-DIAL and Skyline. Overall, we found similar performance with, e.g., lipid profiles that were largely similar between the different tools and across experimental platforms. However, at a more granular level, we also observed systematic differences in the lipids identified due to factors related to the methods and reference databases within the tools in addition to biases introduced through the manual verification of the results. These systematic differences between software tools are not isolated to the analysis of IMS-MS-based lipidomics data; however, the lack of tools in the IMS space increases their impact.

The level of structural detail in lipid annotations is also an important consideration for the interpretation of lipidomics data. Table 2 summarizes the counts of lipid identifications from both software tools, split according to whether the identifications were made at a level that includes individual fatty acid composition (FA) or only sum composition (sum), for all evaluation datasets. A slight majority of lipid identifications produced by these tools include individual fatty acid composition; however, a significant number of lipids were only able to be identified at the level of sum composition, which can hinder the extent of biological interpretation of lipidomics results. The low-detail identifications are likely attributable to analytical limitations (i.e., efficiency of qTOF CID in producing fragments that are useful for identifying fatty acid composition), software limitations, and/or biases introduced through the verification of software results.

Among the most important takeaways from the software evaluations presented in this review is the significant influence of variables, from the experimental conditions through to the validation of results, that have the potential to affect the lipid identifications that are ultimately produced in the analysis of IMS-MS lipidomics data. The impact of some variables, such as the instrumentation or data acquisition methods, are somewhat obvious and have been amply discussed previously [39]. However, factors such as data processing parameters and the experience of the person processing the data and verifying the initial results produced by software tools are less often discussed, despite the significant impact they can have on the reproducibility of results from lipidomics data analysis. The notion that the initial results produced by any data analysis software should not be used uncritically and the requirement that these results be reviewed by an expert prior to interpreting their biological conditions are broadly understood, but the potential for the introduction of bias through this process of manual inspection cannot be ignored. Beyond the implications on the reproducibility of results, manual inspection is also the most labor- and time-intensive portion of lipidomics data analysis and constitutes a significant bottleneck in the analysis of large datasets. Increased efforts are required in the area of informatics for lipidomics data analysis in order to reduce the bias and burden associated with extensive manual inspection and validation of results.

New technological developments involving IMS-MS lipidomics continually create the need for new software tools that facilitate the transition from proof of concept to real-world application. Two areas of particular interest are increasing the depth of structural characterization through the integration of techniques for determining lipid double bond positions (e.g., Paternò-Büchi [40,41], OzID [42,43]) or ultrahigh-resolution IMS separations [44,45] and integrating MS imaging (MSI) with IMS-MS platforms for spatial lipidomics [27,46]. Although the technological details differ significantly, these two development areas face essentially the same challenge with respect to their broader use in practical applications, i.e., the lack of software support. Thus, expanding the coverage of software tools beyond the more conventional methods should be an area of particular focus in future software development for IMS-MS lipidomics.

## Figures and Tables

**Table 1:** Survey of software tools for analysis of IMS-based lipidomics data. Abbreviations: DIA, data-independent acquisition; DDA, data-dependent acquisition; MS1, normal MS (without fragmentation); MS2, tandem MS/MS (includes fragmentation); RT, retention time; CCS, collision cross section; HILIC, hydrophilic interaction chromatography; NA, not applicable.

Software	Supported File Formats	Workflow	Acquisition Modes	Annotation Method	Ref.
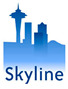	Input: raw files (Agilent, Bruker, Sciex, Waters, Thermo and Shimadzu), mzML, mz5, mzXML Output: csv	Targeted	DDA, DIA	MS2 fragmentation, iRT calibrated RT, CCS experimental library containing 516 unique lipids	[22]
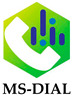 MS-DIAL	Input: raw files (Agilent, Waters and Bruker), must be converted to IBF Output: mztab-M	Untargeted	DDA, DIA	MS2 fragmentation, RT, CCS experimental and predicted library containing 581,047 unique lipids	[23]
LiPydomics	Input: csv (feature table) Output: png, xlsx	Untargeted	DDA, DIA	HILIC RT, CCS experimental and predicted library containing 145,388 unique lipids	[24]
LipidIMMS (Lipid4DAnalyzer)	Input: supports Agilent, Bruker, Waters, Sciex MS1 peak table (.csv format), MS2 data files (.mgf/.msp /.cef format), RT calibration table (.csv format, optional) Output: html, pdf, csv	Untargeted	DDA, DIA	MS2 fragmentation, RT, CCS experimental and predicted library containing 267,716 unique lipids	[25]
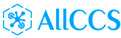	Input: csv Output: csv	Untargeted	DDA, DIA	Experimental and predicted CCS library	[26]
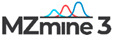	Input: tdf, tsf (Bruker), mzML Output: csv	Untargeted	MS1	User-supplied RT and CCS library	[27]
DEIMoS	Input: mzML Output: csv, mgf, mzML	Untargeted	DDA, DIA	NA	[28]

**Table 2:** Summary of lipid identifications from DTIMS and TIMS evaluation data in positive (+) and negative (−) ionization modes using Skyline and MS-DIAL. Each contains the count of lipid identifications made at levels that include individual fatty acid composition (FA) or only sum composition (sum).

FA		DTIM (+)		DTIM (−)		TIMS (+)		TIMS (−)	
	*sum*								
**Skyline**		149		212		9		32	
			68		11		92		122
**MS-DIAL**		86		36		111		175	
			137		13		91		63

## Data Availability

No new data were created or analyzed in this study. Data sharing is not applicable to this article.
